# Mitosis in Cancer Cell Increases Immune Resistance via High Expression of HLA-G and PD-L1

**DOI:** 10.3390/cancers12092661

**Published:** 2020-09-18

**Authors:** Matti Ullah, Warda Aoudjeghout, Cynthia Pimpie, Marc Pocard, Massoud Mirshahi

**Affiliations:** CAP-Paris Tech., INSERM U1275, Hôpital Lariboisière, 2, rue Ambroise-Paré, 75010 Paris, France; matti.ullah@inserm.fr (M.U.); aoudjeghout.warda@yahoo.com (W.A.); cynthia.pimpie@inserm.fr (C.P.); marc.pocard@inserm.fr (M.P.)

**Keywords:** mitosis, immune resistance, cell cycle, HLA-G, PD-L1

## Abstract

**Simple Summary:**

Cancer results from “aggressive division” of cells, that is unchecked by the immune cells. Cancer cells express several proteins to evade the immune response. One of the main treatment against cancer is to target and block their cell division. We found that, the expression of these proteins is increased during the mitosis stage of cell division. Therefore the blockade of cell cycle in the mitotic phase may results in increased immune resistance that can further help the cancer cells in evading immune attack.

**Abstract:**

Cancer is a result of “aggressive” division and uncontrolled proliferation of the abnormal cells that survive attack by immune cells. We investigated the expression of HLA-G and PD-L1 with the different stages of cancer cell division along with their role in the interaction of immune cells in vitro. Ovarian cancer (OVCAR-3) and chronic myeloid leukemia cell line (K-562) are used for this study. The correlation of protein expression with percentage of cells in each phase (G1, S and G2 phase) was evaluated through FACS. Cells were synchronized in G1, G2 and mitotic phase to evaluate gene (RT-qPCR) and protein expression (FACS). Real-time immune cell attack (RTICA) analysis with PBMCs (peripheral blood mono-nuclear cells) and cancer cells were performed. We found that cells expressing higher levels of HLA-G and PD-L1 are mainly in G2 phase and those expressing lower levels are mainly in G1 phase. Evidently, the higher expression of the two proteins was observed when synchronized in mitotic phase as compared to low expression when synchronized in G1 phase. RTICA analysis showed the presence of HLA-G delayed the lysis of the cells. In conclusion, the cancer cell can escape from immune cells in division stage that suggests the impact of mitosis index for cancer immunotherapy.

## 1. Introduction

Cancer is a result of “aggressive” division and uncontrolled proliferation of abnormal cells. Ideally, the first step in the treatment of cancer is to stop the proliferation of these cells. The classical approach against cancer is to disrupt the cell division cycle disabling the proliferation of cells. Several strategies have been used over recent decades to arrest cancer cell proliferation. Mitotic or microtubule inhibitors (like 5-fluorouracil, vincristine and paclitaxel) have kept a prime place in the therapeutic approach against cancer [1,2,3]. However, with a better understanding of the cell cycle, new approaches have been applied in targeting other phases of the cell cycle. The progression of the cell cycle is a highly regulated step, which is controlled by various cyclin-dependent kinases such as four members of CDK family (1, 2, 4 and 6), and is known to regulate the cell cycle. The discovery of CDKs and their role in cell division cycle enlightened new approaches to target cancer division. Each of the CDK expressions regulate specific cell cycle phases e.g., CDK4/6 governs the progression of cell cycle through G1 phase, CDK2 controls the progression through S-phase while CDK-1 facilitates the cells at G2/M phase to enter mitosis. Targeting these cyclin molecules has led to another therapeutic strategy in blocking the cell cycle. There are up to 10 CDK inhibitor compounds currently undergoing clinical trials. Among these compounds, CDK4/6 inhibitors are of prime importance; two CDK4/6 inhibitors have already been approved by FDA to be used as combination therapy [4,5]. Following the FDA approval of several CDK4/6 inhibitors like abemaciclib and ribociclin, initial clinical trial results have pointed out a cross-talk between cell cycle arrest and immune cell interaction [6,7,8]. Cyclin D-CDK4 complex regulates the PD-L1 expression through proteasome-mediated degradation by Cullin 3^SPOP^ E3 ligase [9]. It has been reported that selective CDK4/6 pharmacologically enhances the tumor infiltration by immune cells [10,11]. Further, the use of anti-PD-L1 therapy improved the overall effect of CDK4/6 inhibitors [12]. Cancer cells exhibit one or several immune checkpoints to evade against immunity. Among several immune checkpoints, CTLA-4 and PD-L1 are the widely studied ones. The PD-L1 is expressed by the tumor cells as well as immune cells such as antigen presenting cells (APCs) and tumor-associated macrophages (TAMs) while CTLA-4 is mainly expressed by activated T-cells such as memory T-cells and regulatory T-cells [13,14,15]. Another protein, HLA-G, has been backed by strong evidence indicating its role in immune suppression [16,17,18,19]. Recently, we have reported that patients with ovarian carcinomatosis express HLA-G that is linked to an increase in numbers of circulating regulatory T-cells and a decrease in number of CD8 cytotoxic T-cells and NK-cells. Moreover, we found that HLA-G is expressed by different cell lines and IL-1β can induce the expression of HLA-G via NF-κB pathway [20]. As PD-L1 and HLA-G, both can be expressed by tumor cells, it would be interesting to evaluate how different phases of cell cycle affect the expression of these two proteins. In this study, we evaluated the expression of the two immune checkpoints in different cell cycle phases and the effect of their presence on interaction of immune cells and cancer cells.

## 2. Material and Methods

### 2.1. Cell Lines

We chose two different cell lines, based on the expression of the two proteins to be studied i.e., HLA-G and PD-L1. An adherent human ovarian cancer cell line (OVCAR-3) and suspension human chronic myeloid leukemia cell line (K-562), both purchased from ATCC, were used. Cell lines were cultured using RPMI-1640 medium (OVCAR-3) and IMDM medium (K-562) supplemented with 10% fetal calf serum, 50 U/mL penicillin-streptomycin and 2 mM L-glutamine in an incubator with a humidified atmosphere at 37 °C containing 5% CO_2_.

### 2.2. Immunofluorescence

OVCAR-3 cells were cultured at 50% confluence in Lab-Tek^®^ 3-well chamber slide for 24 h before fixation for 10 min with 4% paraformaldehyde. The cells were then washed with PBS. Non-specific binding sites were blocked using 1% BSA in PBS-tween (0.1%). Cells were then incubated with primary antibodies, anti-HLA-G (rabbit purchased from Epigentek, Farmingdale, NY, USA) and anti-PD-L1 (mouse purchased from Proteintech, Rosemont, IL, USA), respectively, for 3 h. Then, cells were washed with PBS-Tween (0.1%) and incubated with secondary antibodies (anti-rabbit AF488 and anti-mouse AF594; Invitrogen, Waltham, MA, USA) for 1 h. The incubations were performed at room temperature with mild agitation. The slides were then washed and mounted with DAPI containing aqueous mounting medium by Vectashield^®^ for images to be taken using ZEISS LSM 900 confocal microscope.

### 2.3. FACS Analysis for Cell Cycle and Protein Expression

#### 2.3.1. FACS

For FACS analysis, cells were stained using an indirect staining method. Cells were collected from the flasks (with or without treatment), and were fixed using ice-chilled 70% ethanol at 4 °C. The cells were then washed and incubated with primary antibodies (as mono-staining) overnight at 4 °C at a concentration of 1 µg for HLA-G (Epigentek, USA) and 1.25 µg for PD-L1 (Proteintech, USA) per 100,000 cells. In parallel, the cells were stained with equivalent amount of isotype antibodies (Figure S4) i.e., rabbit polyclonal IgG and mouse monoclone IgG1 antibody (Invitrogen, ThermoFisher Scientific, Waltham, MA, USA). On the following day, the cells were then washed and incubated with secondary antibodies (anti-rabbit AF488 and anti-mouse AF546; Invitrogen) for 2 h. To stain the cells in mitosis, we used MPM2 antibody (Anti-phospho-Ser/Thr-Pro, Cy5 conjugate; Sigma-Aldrich, Saint Quentin Fallavier, France) at concentration of 2 µg per 100,000 cells. The cells were analyzed using 3-Laser Flow cytometer “Gallios” by Beckmann Coulter and the results were analyzed using “Kaluza Analysis 2.1” software. Cell cycle was recorded through DAPI, using Michel H. Fox algorithm (built-in Software).

#### 2.3.2. FACS Analysis

For MFI (mean fluorescence intensity), mode fluorescence intensity was evaluated as it expressed the fluorescence of the majority of the cells. Further, for pre-screening, the fluorescence peak was gated in ten equal parts termed as deciles (marked as A to J with 10% of cells in each gate) in increasing intensities of fluorescence (Figure S2), so that cells in 1st decile expressed the least intensity of the proteins and 10th decile represented cells that expressed the highest protein levels. The cell cycle using DAPI was determined for each decile, and correlation was evaluated for protein level using mean fluorescence intensity of each gated region (Figure S2).

### 2.4. Cell Synchronization

Cell lines were synchronized in G1 phase, G2 phase and Mitotic phase using lovastatin [21], CDK-1 inhibitor (RO-33022 by Sigma Aldrich) [22] and nocodazole (mitotic inhibitor) [23]. The synchronizing agents were dissolved in DMSO, therefore DMSO-treated cells were used as control. Each of the cell lines was treated for 24 h with the agents to synchronize cells in a specific phase. The concentration (as µg/mL for 2 million cells in each condition) is given in the Table 1 for each cell line.

### 2.5. mRNA Expression of HLA-G and PD-L1 via qPCR

RNA was isolated from the cells using ReliaPrep™ RNA Miniprep Systems by Promega following the treatment with cell cycle inhibitors. The RNA was reverse-transcripted to cDNA using Maxima First Strand cDNA Synthesis Kit. The cDNA was then used at a concentration of 2.5 ng/10 uL reaction in LightCycler 96 by Roche using SYBR green master mix in 3-step amplification. The relative expression was measured through ∆∆CT method using beta-actin as housekeeping gene. The primer sequences were: beta-actin (sens: AGA GCT ACG AGC TGC CTG AC); anti-sense: AGC ACT GTG TTG GCG TAC AG), HLA-G (sens: GCG GCT ACT ACA ACC AGA GC; anti-sense: GAG GTA ATC CTT GCC ATC GTA G), PD-L1 (sens: CAA AGA ATT TTG GTT GTG GA; anti-sense: AGC TTC TCC TCT CTC TTG GA) [24,25].

### 2.6. Real-Time Immune Cell Attack (RTICA) Analysis

In order to determine the effect of HLA-G and PD-L1 on interaction of immune cells with cancer cells, we co-cultured OVCAR-3 (stained using NucBlue Live ReadyProbes™, ThermoFisher Scientific) with PBMCs in 5:1 ratio in RPMI medium with or without HLA-G (3 ng/mL) and PD-L1 (3 ng/mL). We performed cinematography by taking pictures at interval of 30 s. The PBMCs were collection from human blood following Ficoll separation protocol (oral consent taken). In parallel, we used cells synchronized in G1 phase (lovastatin) and mitotic phase (nocodazole). The images were analyzed using Image-J. The cell was considered lysed as soon as cytoplasm started leaking and the immune cells were considered attached if their attachment remained constant for the 10 frames i.e., 5 min (Supplementary Video provided; Video S1; for unsynchronized cells in normal culture medium).

### 2.7. Statistical Analysis

The statistical analysis was performed using GraphPad Prism 6. One-way ANOVA (post-Hoc Tukey’s test) was performed to compare the difference between the two groups, while correlation was calculated as spearman correlation.

## 3. Results

### 3.1. HLA-G and PD-L1 Expression in Dividing and Non-Dividing Cells

**Figure 1:**
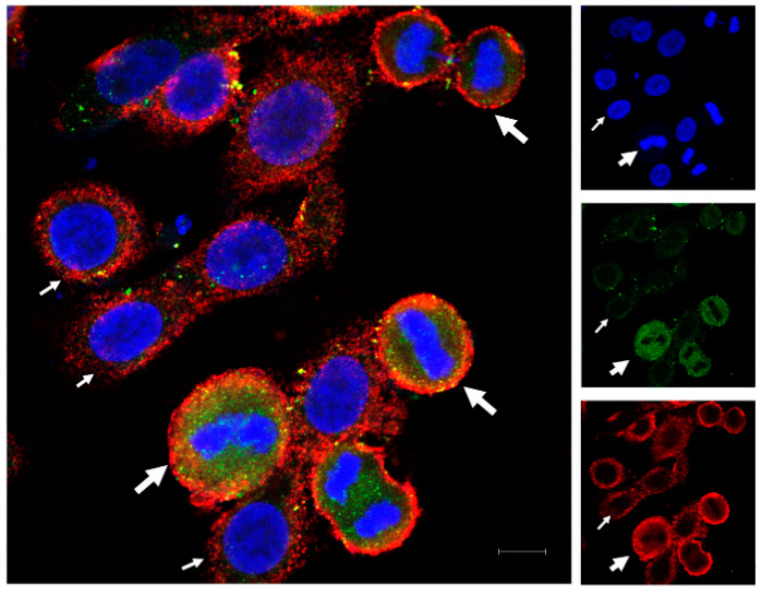
Immunofluorescence for HLA-G and PD-L1 in OVCAR-3 cells. OVCAR-3 cells were cultured in cell chamber and stained for HLA-G (green) and PD-L1 (red) and DAPI (blue) for nucleus. An increased expression of the two proteins (HLA-G and PD-L1) was observed in dividing cells (thick white arrows with dividing nuclear material) compared to non-dividing cells (thin white arrows; scale bar: 10 µm).

The immunofluorescence for HLA-G and PD-L1 in OVCAR-3 cells showed variable expression of the two proteins in monotype cell line. Some of the cells expressed higher protein intensity compared to other cells. We found that the dividing cells expressed higher protein expression for HLA-G as well as PD-L1 compared to non-dividing cells (Figure 1), and this increase in expression varies through different mitotic stages (Figure S1). We found the high HLA-G expression among the cells in prophase, which is the starting point of cell division. This expression remains high until the telophase. However, the expression of HLA-G decreases in cytokinesis, although it still remains high as compared to non-dividing cells. This suggests that the HLA-G expression is increased during mitosis but starts to decrease again at the end of the mitosis. Further, we found that the PD-L1 expression was high throughout the mitotic stages as well as in cytokinesis as compared to non-dividing cells.

### 3.2. Pre-Screening through FACS

**Figure 2:**
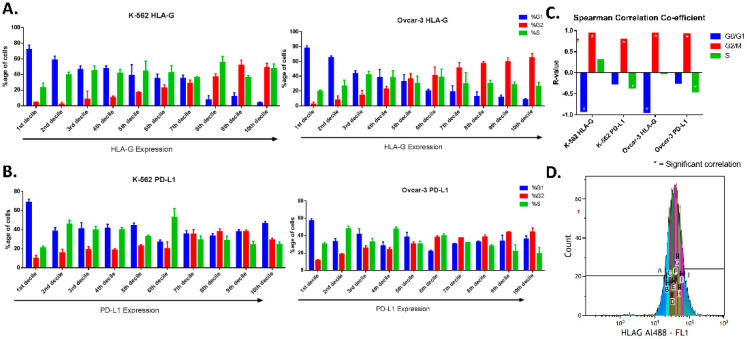
Cell cycle phases based on expression of HLA-G and PD-L1 (FACS). OVCAR-3 and K-562 cells were stained for HLA-G (a) and PD-L1 (b). Cell cycle analyzed after gating protein expression peak in 10 equal parts (deciles; A-J) in increasing protein expression. (**A**). For HLA-G, both the cell lines showed that the cells present in first deciles (low expression) were mainly in G0/G1 phase, while those in higher deciles have the majority of the cells in G2 phase. (**B**). For PD-L1, The G0/G1 and S-phase cells showed similar percentage of cells present in each phase among different gates (from low to high expression). The G2 phase cells were present more in higher intensity gates compared to lower ones. (**C**). The FACS analysis showed strong positive correlation of G2 phase (r_s_ > 0.9) with HLA-G and PD-L1 in both the cell lines, while strong negative correlation of G0/G1 phase was found only for HLA-G (r_s_ > 0.9) but not with PD-L1. The S-phase did not show any correlation for HLA-G with either of the cell lines, and weak negative correlation (r_s_ = −0.3) was found for PD-L1. (**D**). An image of the peak expression gated for 10 deciles marked as A to J with increasing expression of the protein.

The cells (using expression intensity peak) were gated in deciles with increasing fluorescence expression (Figure 2D, Supplementary Figure S2). The cell cycle was analyzed for each gated cell to find out the correlation between cell cycle phase and expression intensity. We found the increasing percentage of cells in G2 while there was a decreasing percentage of cells in G0/G1 phase from 1st decile (low expression) to 10th decile (high expression) for HLA-G in both the cell lines (Figure 2A). The expression intensity of HLA-G correlated positively with percentage of cells in G2 (r_s_ = 0.93 for K-562 and r_s_ = 0.94 for OVCAR-3) while there was a negative correlation with the percentage of cells in G0/G1 phase (r_s_ = −0.91 for K-562 and r_s_ = −0.93 for OVCAR-3). This shows that majority of cells in low or intermediate expression of HLA-G were present in G0/G1 phase while the majority of cells that show high expression of HLA-G were present in G2 phase (Figure 2C). There was no significant difference found between percentage of S-phase cells from 1st decile to 10th decile and no correlation was observed for percentage of S-phase with expression of HLA-G.

For PD-L1, we did not find any regular pattern for G0/G1 and S-phase (Figure 2B). Although the cells with lowest expression of PD-L1 (first and second deciles) consisted mostly of G0/G1 phase (near 60%). Moreover, very low percentage of G2 cells (less than 20%) were present in low expressing cells, while majority of the G2 phase cells showed high expression of PD-L1. No Significant correlation was found for G0/G1 phase with expression intensity of PD-L1, while G2 phase showed strong positive correlation (r_s_ = 0.79 for K-562 and r_s_ = 0.92 for OVCAR-3). For PD-L1, S-phase showed weak negative correlation (r_s_ = −0.36 for K-562 and r_s_ = −0.45 for OVCAR-3) with PD-L1 expression intensity (Figure 2C).

### 3.3. Cell Synchronization

**Figure 3:**
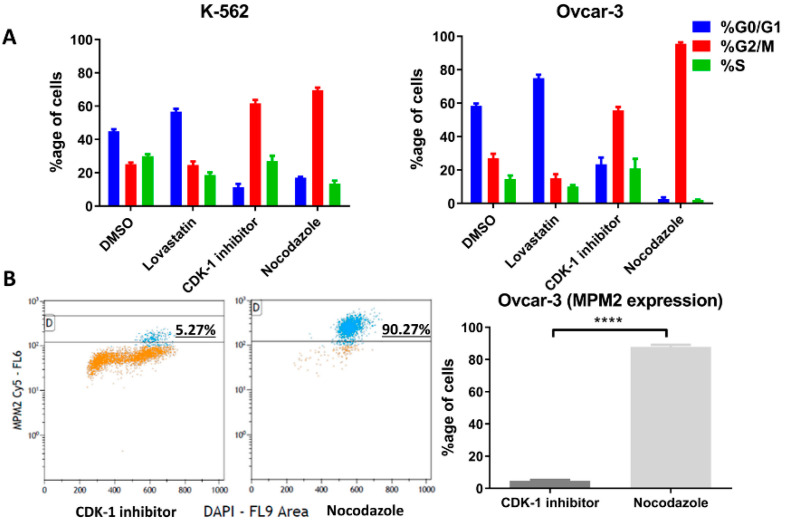
Cell phase following cell cycle inhibition. The two cell lines were synchronized in G1 phase using lovastatin, G2 phase using CDK-1 inhibitor and mitosis phase using nocodazole. (**A**). Percentage of each cells in different cell cycle phases (K-562, right and OVCAR-3 left) analyzed through FACS (Michael H. Fox algorithm) using DAPI is presented. (**B**). Percentage of positive cells with MPM2 staining show mitotic cells (OVCAR-3) following CDK-1 inhibitor and nocodazole treatment. Nocodazole blocks cells in mitotic phase while low expression of MPM2 in CDK-1 inhibitor-treated cells shows that it blocks cells only in G2 phase (****, *p* < 0.0001 unpaired *t*-test).

The cells were treated with lovastatin, CDK-1 inhibitor and nocodazole to synchronize in G1, G2 phase and mitotic phase, respectively. DMSO-treated cells were used as control unsynchronized cells. Figure 3A shows the percentage of cells in three different phases after treatment. For OVCAR-3 cells treated with nocodazole, only non-adherent cells were collected to further enrich mitotic cells. Further, we performed FACS analysis using MPM2 staining (Figure 3B) in order to determine the mitotic index for CDK-1 inhibitor- and nocodazole-treated cells. We found that CDK-1 inhibitor-treated cells have very low mitotic index (Figure 3B) with percentage of MPM2 positive cells (4.6 ± 0.6) compared to those treated with nocodazole (87.8 ± 1.3). Therefore, CDK-1 inhibitor-treated cells were majorly in G2 phase while nocodazole blocked cells in mitotic phase.

### 3.4. HLA-G (Protein and Gene) Expression in Synchronized Cells

**Figure 4:**
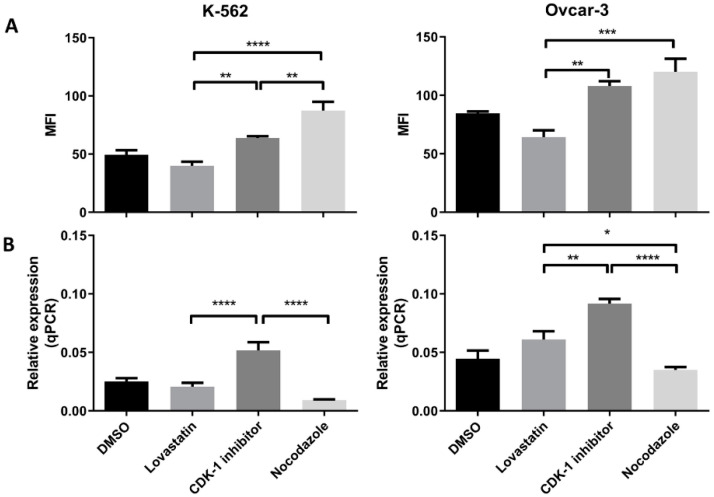
HLA-G (protein and mRNA) expression by K-562 and OVCAR-3. (**A**). Mean fluorescence intensity (MFI) using mode fluorescence intensity is shown for the two cell lines. DMSO-treated cells are shown as a control with unsynchronized cells. Nocodazole-treated cells have the highest expression of HLA-G protein in each of the cell lines while lovastatin-treated cells showed the lowest expression, not different to unsynchronized cells. The CDK-1 inhibitor-treated cells showed intermediate expression for K-562 cells while for OVCAR-3 cells, expression was higher than lovastatin-treated cells but not different than nocodazole-treated cells. (**B**). qPCR analysis showed that both cell lines express higher gene expression when treated with CDK-1 inhibitor (G2 phase) compared to lovastatin (G1) and nocodazole (mitotic)-treated cells. No difference was observed for gene expression in lovastatin (G1)-treated cells compared to nocodazole (mitotic)-treated cells in K-562 cell line. However, OVCAR-3 cells showed the lowest mRNA expression in nocodazole-treated cells that was significantly lower than lovastatin-treated cells (One-Way ANOVA; Tukey’s test * < 0.05, ** < 0.005, *** < 0.001, **** < 0.0001).

Both the cell lines when synchronized in different phases, showed difference in the HLA-G expression (protein as well as mRNA expression). In K-562 cells (Figure 4A) we found that the lovastatin-treated cells showed lowest expression of the HLA-G protein compared to CDK-1 inhibitor- (*p* < 0.005) and nocodazole-treated cells (*p* < 0.0001). However, there was no significant difference in expression compared to unsynchronized cells. Further, the nocodazole-treated cells showed even higher expression compared to CDK-1 inhibitor-treated cells (*p* < 0.005). However, the gene expression for HLA-G in K-562 cell lines (Figure 4B) was only increased in CDK-1 inhibitor-treated cells (*p* < 0.0001 compared to lovastatin and nocodazole. There was no significant difference found for gene expression of HLA-G between unsynchronized cells, lovastatin-treated cells and nocodazole-treated cells.

Similar results were observed for OVCAR-3 cell lines (Figure 4A). The lovastatin-treated cells showed lowest expression compared to CDK-1 inhibitor- (*p* < 0.005) and nocodazole (*p* < 0.001)-treated cells. In contrast to K-562 cell line, there was no significant difference found between CDK-1 inhibitor-treated cells compared to nocodazole-treated cells. This shows that the HLA-G expression in OVCAR-3 cell lines is increased during G2 phase and does not change during mitosis. Moreover, similar to K-562 cell lines, we found that the gene expression (Figure 4B) was highest in CDK-1 inhibitor-treated OVCAR-3 cells compared to lovastatin- (*p* < 0.005) and nocodazole (*p* < 0.0001)-treated cells. In addition, the lovastatin-treated cells showed increased gene expression of HLA-G compared to nocodazole-treated cells (*p* < 0.05).

### 3.5. PD-L1 (Protein and mRNA) Expression in Synchronized Cells

**Figure 5:**
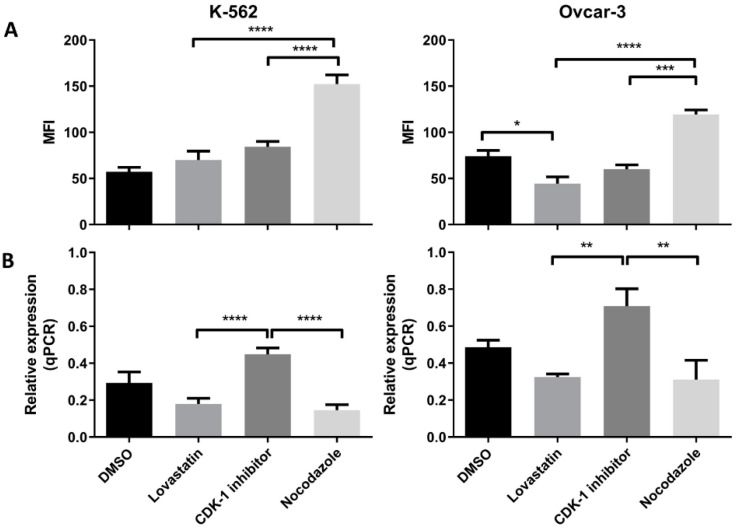
PD-L1 (protein and mRNA) expression in K-562 and OVCAR-3. (**A**). MFI using mode fluorescence intensity is shown for the two cell lines. Both the cell lines showed significant increase in PD-L1 protein expression in the cells treated with nocodazole (mitotic), no difference was observed between lovastatin- (G1) and CDK-1 inhibitor (G2)-treated cells. (**B**). Similar gene expression to HLA-G was found for PD-L1 where G2 phase cells had higher gene expression compared to cells treated with lovastatin (G1) and nocodazole (mitotic), while there was no difference found in the gene expression between cells treated with lovastatin (G1) and nocodazole (Mitotic). (One-Way ANOVA; Tukey’s test * < 0.05, ** < 0.005, *** < 0.001, **** < 0.0001).

We found an increased PD-L1 protein expression when cells were treated with nocodazole for both the cell lines (Figure 5A). We found a significant low protein expression in CDK-1 inhibitor-treated cells as compared to nocodazole (*p* < 0.005) in both the cell lines. The lovastatin-treated cells showed similar expression to CDK-1 inhibitor-treated cells, both significantly lower than nocodazole-treated cells. Interestingly, lovastatin-treated OVCAR-3 cells showed significantly lower expression of PD-L1 compared to unsynchronized cells (DMSO), suggesting that cells, when synchronized in G1 phase, show lower expression of PD-L1. In contrast, No change was observed for lovastatin-treated K-562 cells as compared to unsynchronized cells.

We found the highest gene expression of PD-L1 in CDK-1 inhibitor-treated cells for both of the cell lines (Figure 5B). Lovastatin and nocodazole showed significantly lower gene expression of PD-L1 in both the cell lines when compared to CDK-1 inhibitor-treated cells (*p* < 0.0001 for K-562 and *p* < 0.005 for OVCAR-3). There was no significant difference found between lovastatin- and nocodazole-treated cells.

### 3.6. HLA-G and Mitotic Blockade Suppress the Immune Cell Attack

**Figure 6:**
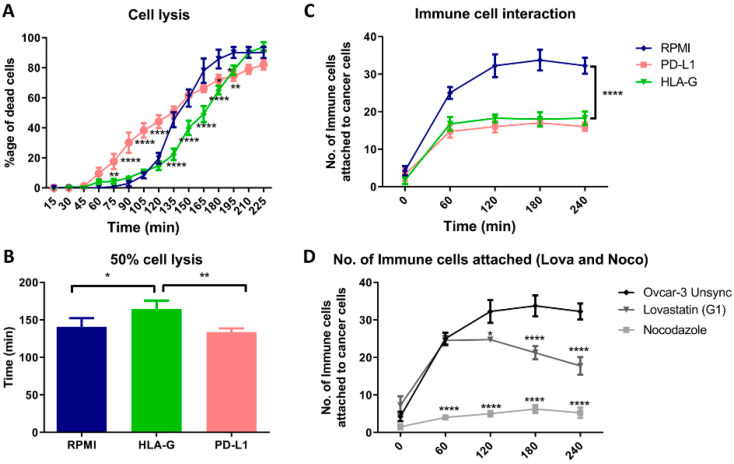
Co-culture of OVCAR-3 cells with PBMCs. (**A**). Time-dependent lysis of OVCAR-3 cells in RPMI medium compared to presence of HLA-G and PD-L1. PD-L1 initially increases the lysis, while HLA-G resulted in delayed lysis of the cancer cells compared to OVCAR-3 cultured in absence of two proteins. (**B**). HLA-G increased the time required for the lysis of half of the population, but no difference with PD-L1. (**C**). PD-L1 and HLA-G, both decreased the attachment of the immune cells as a decrease in total number of cells attached to the cancer cells. (**D**). We found that the interaction between immune cells and mitotic cells (nocodazole) was significantly low compared to G1-synchronized cells (lovastatin) or unsynchronized cells.

When we cultured OVCAR-3 cells with PBMCs in presence of HLA-G and PD-L1, we found that presence of PD-L1 initially resulted in increased lysis of OVCAR-3 cells. While the presence of HLA-G delayed the lysis of the cells (Figure 6A). The statistical analysis showed that HLA-G significantly increases the time required for the lysis of half of the cancer cells by 30 min while there was no difference found in the presence of PD-L1 compared to regular culture medium (RPMI; Figure 6B). Hence, presence of HLA-G in-vitro, provided a better immune protective effect than PD-L1 to the cancer cells. Moreover, when we analyzed the interaction of the immune cells, there was less interaction found between immune cells and cancer cells in presence of HLA-G as well as PD-L1 (Figure 6C). Additionally, this decreased interaction between cancer cells and immune cells was found when we cultured cells synchronized in mitotic phase (Figure 6D; Figure S3). However, the interaction with lovastatin-treated cells (G1) was significantly higher compared to nocodazole-treated cells (mitosis).

## 4. Discussion

Cancer cells express PD-L1 and other immune checkpoints to protect themselves against the immune system. Among these, the PD-1/PD-L1 pathway is one of the most widely studied immune checkpoints expressed by cancer cells. Another newly recognized immune checkpoint is HLA-G, which was initially discovered at maternal–fetal interface with a function to protect the fetus against maternal immune system. CDK 4/6 inhibitors that block cells in G1 phase have been shown to enhance immune cells’ infiltration of tumors [11]. Therefore, it would be important to study the expression of PD-L1 as well as HLA-G in different cell cycle phases to evaluate how the cell division cycle affects the expression of these two immune checkpoints. We used two different cell lines i.e., OVCAR-3 which is adherent human ovarian cancer cell line and K-562 human leukemia suspension cell line. The cell lines were chosen based on the expression of the two proteins i.e., HLA-G and PD-L1.

We found the difference in expression of the two proteins (HLA-G and PD-L1) among the cells of the monoclonal cell line, OVCAR-3. Immunofluorescence analysis showed that the cells in mitotic phase express higher levels of HLA-G and PD-L1 compared to non-dividing cells. In addition, the expression is not uniform throughout the mitosis, rather it changes through different mitotic stages, with HLA-G expression decreasing at the end of the mitosis and the start of the cytokinesis while PD-L1 levels increase in cytokinesis as compared to mitotic phase. This suggests that cancer cells can be more resistant to immune attack during division as compared to the resting stage. It has been reported that PD-L1 knockout does not affect the tumor growth in vivo, therefore the increased expression of PD-L1 can be seen as an additional immune suppressive mechanism adapted by the cells, that further need to be studied [15].

Moreover, we performed FACS analysis (in the two cell lines K-562 and OVCAR-3), where we gated the unsynchronized cells based on their protein expression and analyzed the cell cycle after culturing cells in their regular culture conditions. We found that the cells express higher levels of HLA-G near or during division as the majority of cells that have higher protein levels were mainly in G2 phase as compared to ones with low expression that were found to be in G0/G1 phase. However, the positive correlation of PD-L1 expression only increases in G2 phase. This confirms the previous observation conducted in OVCAR-3 through immunofluorescence in both the cell lines confirming that both HLA-G and PD-L1 expression increases near cell division.

Further, to confirm these initial findings, the expression (protein as well as mRNA) was analyzed following cell synchronization in different phases of cell division. Lovastatin was used to arrest cells in G1 phase, CDK-1 inhibitor to block in G2 phase and nocodazole to block the cells in mitotic phases. DMSO-treated cells were used as control unsynchronized cells as each of the synchronizing agents was dissolved in DMSO. High expression of MPM-2 in nocodazole-treated cells compared to CDK-1 inhibitor-treated cells revealed that CDK-1 inhibitor blocks cells in G2 phase while nocodazole blocks cells in mitotic phase. We found a significant increase in protein expression of HLA-G as well as PD-L1 in cells arrested in mitotic phase confirming that mitotic cells express higher levels of the two proteins as compared to other two phases. Moreover, the OVCAR-3 cell lines showed increase in expression of HLA-G even in G2 phase. However, the expression of HLA-G in K-562 cell line was significantly lower in G2 phase compared to mitotic phase suggesting that although the cells show higher expression in mitotic phase, the increase in G2 phase is cell line dependent. We found that the cells arrested in G1 phase showed lowest expression of HLA-G compared to cells arrested in G2 phase and mitotic phase. Further, the significant lower expression of PD-L1 in OVCAR-3 for lovastatin-treated cells, compared to unsynchronized cells, suggests that cells in G1 phase show low expression of PD-L1. This may be the possible reason for CDK4/6 inhibitors enhancing anti-tumor immunity, as the cells in G1 phase show lower expression of both the immune suppressive proteins [6]. However, this decreased expression can be cancer type-dependent as we did not observe such difference in K-562 cell line. Moreover, we found that among different isoforms of HLA-G, only the sHLA-G1 isoform was expressed by the two cell lines, confirmed through intracellular MEM-G9 staining. We did not find the presence of membrane-bound HLA-G or sHLA-G5 in both of the cell lines (results not shown).

Further, we found that the gene expression for each protein in both the cell lines was higher in G2 phase compared to G1 and mitotic phase. This increased transcription may be translated to higher protein expression in mitotic phase. Further, the decreased transcription in mitotic phase can be the result of a feedback mechanism, and may be responsible for decreased protein expression in G1 phase; however, this needs to be further explored.

In order to see how this altered expression, affects the immune cell interaction with tumor cells, we co-cultured PBMCs with OVCAR-3 cells and filmed their interaction. This co-culture technique has been used to evaluate the inhibitory effect of cancer cells on T-cell activation and their cytokine secretion [26]. However, we developed a real-time attack assay by observing that the immune cells interaction can be evaluated through cinematography. Firstly, we performed this assay in presence of HLA-G and PD-L1 and we found that both HLA-G and PD-L1 can block the interaction of immune cells with cancer cells, however, in-vitro HLA-G provided stronger immune protection to cancer cells compared to PD-L1 that initially resulted in increased lysis of the cancer cells. Similarly, the cells synchronized in mitotic phase showed decreased interaction with immune cells compared to G1 synchronized or unsynchronized cells. The cell lysis analysis was not reliable for the synchronized cells as the release from the synchronization itself resulted in apoptosis. These results suggest that the cancer treatment that results in mitotic blockade may result in lowering the immune cells interaction with cancer cells through increased expression of HLA-G as well as PD-L1.

Although, the increased expression of PD-L1 in mitosis has already been reported [9], our study suggests that use of the mitotic inhibitor cells may result in increased immune resistance in cancer cells. The cells that do not undergo apoptosis during mitotic inhibition therapy may not be susceptible to attack by the immune system, hence resulting in cancer relapse. Further, we can explain the anti-tumor response of anti-PD-L1 with the use of CDK4/6 inhibitors owing to decreased expression of PD-L1 in G1 phase (that synergizes with anti-PD-L1 therapy). However, the underlying mechanism needs to be further explored. Moreover, based on the finding by Deng et al. [11], we suggest the use of anti-HLA-G therapy to compare the results with anti-PD-L1 therapy in combination with CDK4/6 inhibitors.

Finally, proliferation index of tumor (Ki-67 index) is found in correlation with mitotic index in differentiated tumors that helps identify low and high grade tumors [27]. Furthermore, we have shown here that HLA-G and PD-L1 increase during mitosis; these findings suggest that Ki-67 may also be used as a marker of immune resistance in tumor nodules. Recently, Patel et al. showed that high-grade tumors that have higher levels of Ki-67 are more responsive to immune therapies like anti-PD-L1 or anti-CTLA-4 [28]. Therefore, further studies taking these findings in account may be helpful in pre-screening of the cancer patients for immune therapy. In conclusion, our results indicate that cancer cells in a mitotic state resist against immune cell attack via higher expression of immune checkpoint molecules, suggesting the impact of the mitosis index for cancer immunotherapy.

## 5. Conclusions

In conclusion, the cancer cells in mitosis resist to immune cells attack. This phenomena is highly dependent on HLA-G expression during cell division. These results suggest the use of proliferation index (Ki-67), which is directly related to mitotic index, as a marker of immune resistance. In addition, the use of mitotic inhibitors may result in increased immune resistance, therefore should be avoided. However, the cells in G1 phase express lower levels of HLA-G as well as PD-L1, therefore cell cycle inhibitors that block cells in G1 phase should be preferred to lower immune resistance.

## Figures and Tables

**Table 1:** Concentrations (µg/mL) of synchronizing agents

Synchronising Agent	Ovarian Cancer (OVCAR-3)	Chronic Myeloid Leukemia Cell Line (K-562)
Lovastatin	32	27
CDK-1	9	5
Nocodazole	0.48	0.4

## Supplementary Materials

The following are available online at https://www.mdpi.com/2072-6694/12/9/2661/s1, Figure S1: Immunofluorescence for HLA-G and PD-L1 protein expression, Figure S2: Explanatory figure for cell cycle analysis based on decile 2 (Figure 2), Figure S3: Images captured at 15 min after co-culture of BMNCs (Plain arrow; small round cells) with OVCAR-3 cells (Dotted Arrow; with stained Nuclei in blue), Figure S4: FACS overlay peaks for Isotype and specific staining, Video S1: OVCAR-3 cell were co-cultured with BMNCs as explained in materials and methods.
