# Supplementary material for: Mitosis in Cancer Cell Increases Immune Resistance via High Expression of HLA-G and PD-L1
Source: Cancers (Basel). 2020 Sep 18;12(9):2661. doi: 10.3390/cancers12092661 (PMC7564851; doi:10.3390/cancers12092661)
Supplement: Supplementary file 1 [file cancers-12-02661-s001.zip › cancers-932734-suppl-Proofreading/cancers-932734-suppl.docx]

Article

Mitosis in Cancer Cell Increases Immune Resistance via High Expression of HLA-G and PD-L1

Matti Ullah, Warda Aoudjeghout, Cynthia Pimpie, Marc Pocard and Massoud Mirshahi

Supplementary Materials:


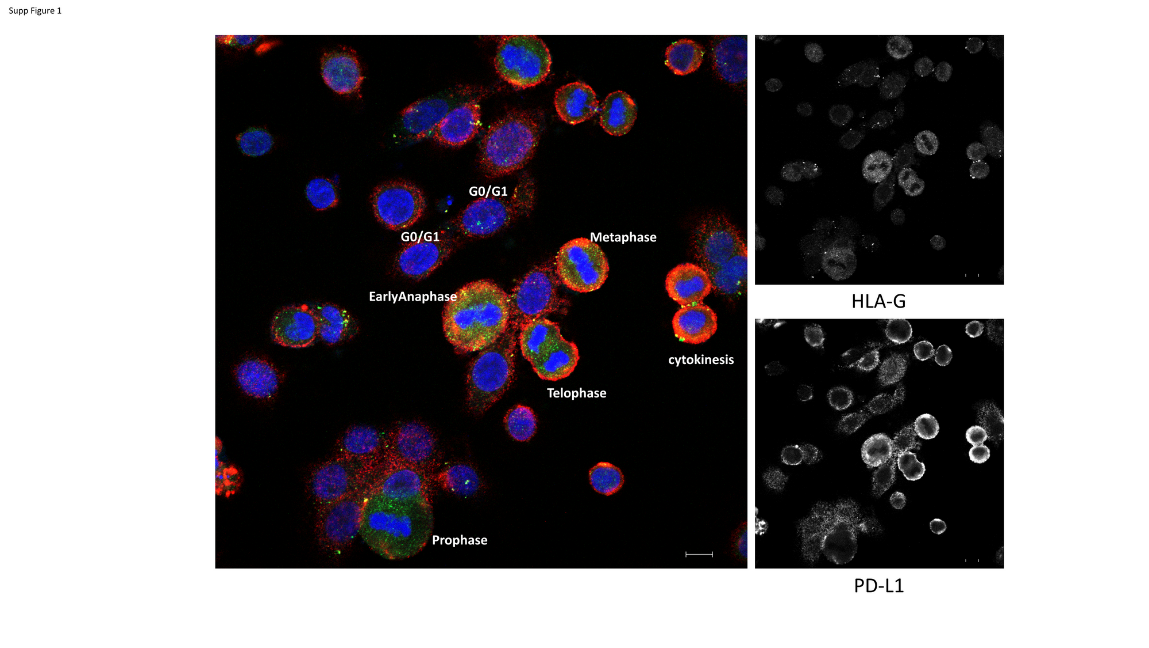


**Figure S1.** Immunofluorescence for HLA-G and PD-L1 protein expression. Cells are shown in different mitotic phases based on the visual analysis of DNA arrangement and cell membrane division. It can be seen that HLA-G and PD-L1 expression increases when the nuclear material starts to divide i.e., prophase. However, during the cytokinesis (where the cell membrane is dividing after completion of mitosis) the HLA-G expression again decreases. But, the expression of PD-L1 was even increased more in cytokinesis phase (Scale bar: 10µm).A


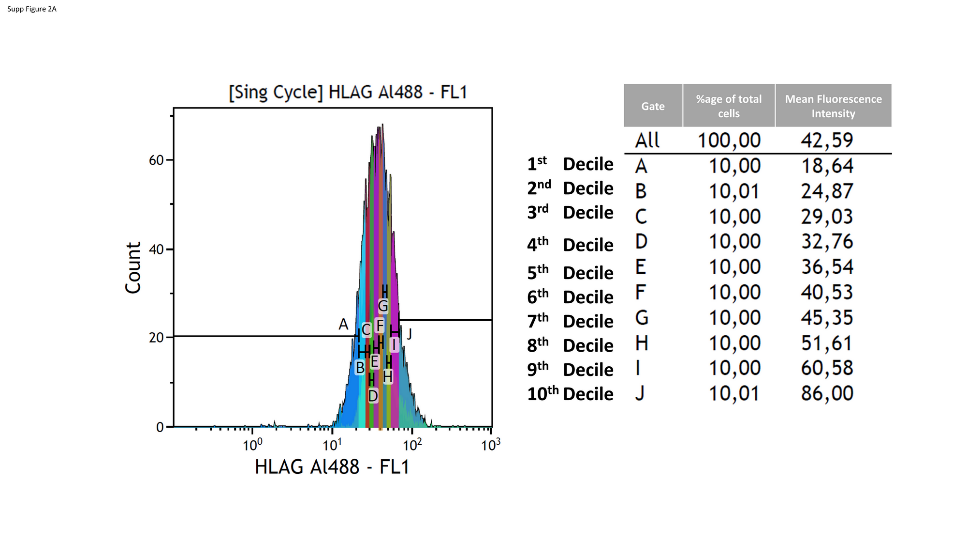


B


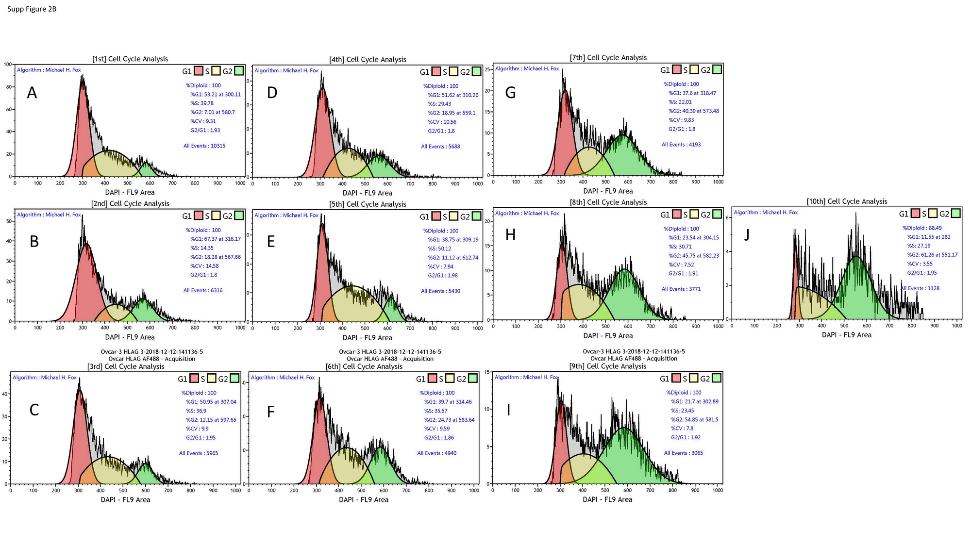


C


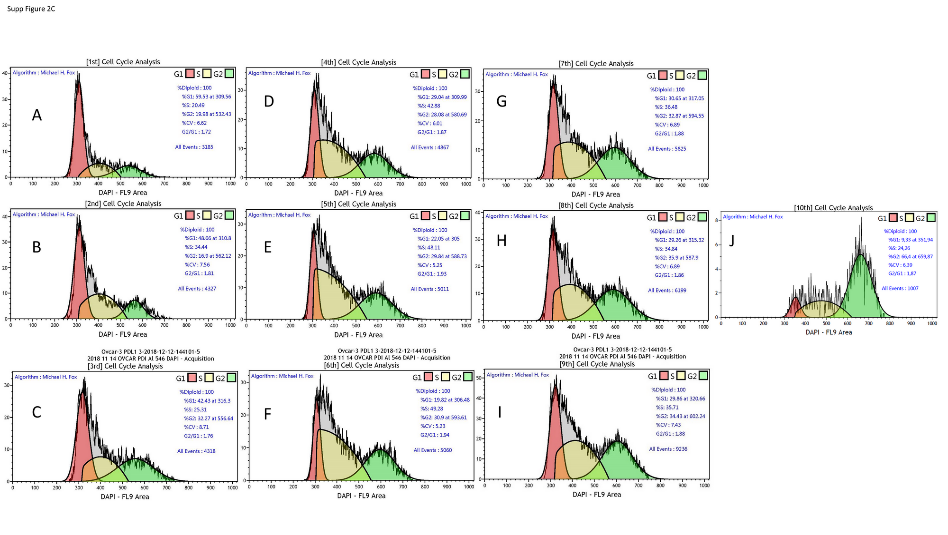


**Figure S2.** Explanatory figure for cell cycle analysis based on decile 2 (Figure 2). (**A**). The expression Peak from FACS was gated for each cell line to have equal number of cells in increasing protein expression manner. The cells present in 1^st^ Decile, shown as A in the peak are the group of cells with lowest expression while that of 10^th^ decile (marked as J) are the one with the highest expression of the proteins as mean fluorescence intensity shown in table for each decile. Cell cycle analysis was done for each gated cells and shown in Figure 2. (**B**). The cell cycle expression of HLA-G expressing cells gated for different expression (A-J) evaluated using Michael H. Fox Algorithm. (**C**). The cell cycle expression of PD-L1 expressing cells gated for different expression (A-J) evaluated using Michael H. Fox Algorithm.


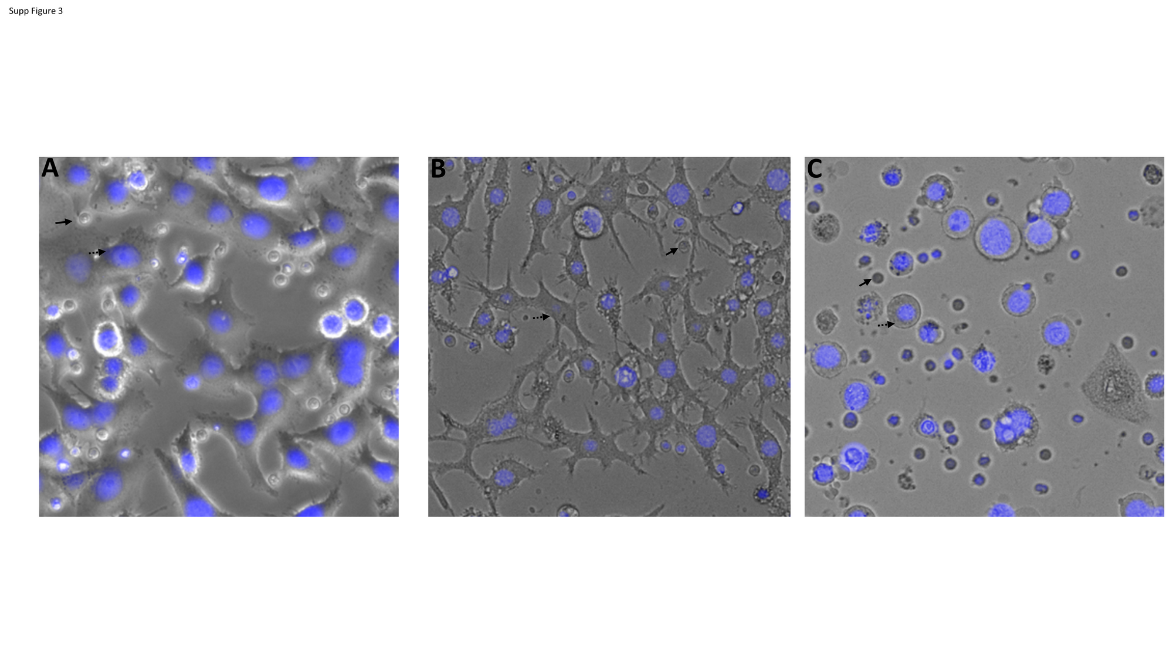


**Figure S3.** Images captured at 15 minutes after co-culture of BMNCs (Plain arrow; small round cells) with OVCAR-3 cells (Dotted Arrow; with stained Nuclei in blue). (**A**). Unsynchronized cells. (**B**). Cells treated with Lovastatin (G1-phase) for 24 hours. (**C**). Cells treated with Nocodazole (Mitotic cells) for 24 hours.


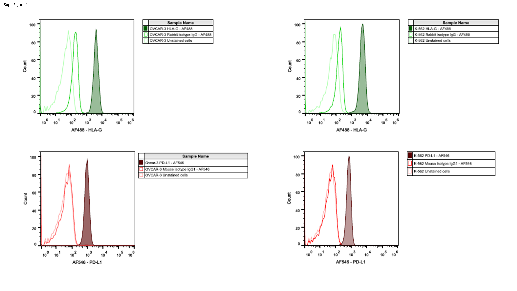


**Figure S4.** FACS overlay peaks for Isotype and specific staining. The FACS peak for isotypes (Top: HLA-G and Bottom: PD-L1) are shown as empty peaks in dark color while the solid color peaks show the expression of the two cell lines (Left: OVCAR-3 and Right: K-562).

**Video S1:** OVCAR-3 cell were co-cultured with BMNCs as explained in materials and methods. The video shows the attack and lysis of the unsynchronized cells in regular medium condition

| 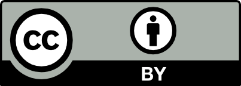 | © 2020 by the authors. Submitted for possible open access publication under the terms and conditions of the Creative Commons Attribution (CC BY) license (http://creativecommons.org/licenses/by/4.0/). |
| --- | --- |
